# The Story of the Insane from Year to Year

**Published:** 1902-02-01

**Authors:** 


### THE STORY OF THE INSANE FROM YEAR TO YEAR.

Fourteenth Annual Series.

DORSET COUNTY ASYLUM.

On January 1st, 1900, this asylum contained 707 patients, of whom 569 were chargeable to the county, 99 were private patients, and 39 were chargeable to unions in other counties. On the last day of the year the numbers had risen to 718, and the increase was almost confined to the county. There were 100 first admissions, 16 readmissions, 39 discharged recovered, 4 discharged relieved, 17 not improved, and 45 deaths. The average number resident was 716, and the total number under treatment was 819. Calculated on the admissions, and excluding transfers, the recoveries stand at 39·39 per cent., and, calculated on the average number resident, the deaths stand at 6·28 per cent. The recoveries are therefore about the average in county asylums, while the deaths are much under the average. On referring to Table X we find that such moral causes as domestic trouble, anxiety and religion are supposed to have been the causes of insanity in 24 of the year's admissions, and among the physical causes hereditary influence comes first with 36 cases, congenital defect with 14, previous attacks with 15, and intemperance in drink has only 2 cases credited to it. Of the deaths, cerebral and spinal diseases account for 12, consumption for 5, and pneumonia and other lung diseases for 7. Not fewer than 28 of the 39 recoveries had been less than a year in the asylum, 6 had been under two years, and one recovered who had been nearly 10 years resident. The asylum is full or rather more than full and additions are to be carried out immediately, and as the asylum adds about 10 every year to its store of pauper lunatics, it is evident that these additions ought not to accommodate fewer than 200 patients. It is a curious fact that the increase in the number of insane throughout England is practically confined to so-called paupers; and Dr. MacDonald thinks this may be accounted for by the county asylums becoming better known, and by the advantages of treatment therein being more appreciated. These statements are unquestionably correct; but they really show that a non-pauper class is availing itself of these advantages; and it is unfortunately only too true that the independence of that class has been sapped to a great extent by the temptation held out to them. Indeed there was almost no alternative, for the charitable lunatic hospitals looked out for patients well able to pay; the private asylum charges were prohibitive; there were no annexes for private patients at our county asylums, and so the class we speak of was gradually driven to enroll itself among the paupers. We congratulate Mr. Gibbons, the head attendant, on having received a suitable pension. The visitors say that "Dr. MacDonald continues to take the liveliest interest in the welfare of the asylum," and "that he is most efficiently supported by Dr. Davidson." The weekly rate of maintenance is 9s. 4d. for home patients, and 14s. for out-county cases. Private patients pay from 10s. 6d. to 42s.

### TEZPUR LUNATIC ASYLUM, ASSAM.

AT the beginning of the year this asylum contained 119 patients, and on the last day of December the number had fallen to 114. There were 30 admissions, 14 discharged recovered, and 17 deaths. The daily average number resident was 122, and the percentage of deaths on this was 13·86. Calculated on the admissions the recoveries stand at 45·16 per cent. Compared with English asylums, both these percentages are high; but, as has often been pointed out, the statistics of a single year, even in a large asylum, are not of much use for purposes of comparison, and in a small asylum they are of no use at all. Table VII. shows the alleged causes of insanity in all of the patients resident during the year 1900, and from it we learn that ganja smoking is responsible for the disease in 21 cases, the use of bhang in 1 case, opium eating in 3, spirit drinking in 2, fever in 4, epilepsy in 10, and hereditary influence in 4. Various moral causes account for 7, while 93 were of unknown origin. The latter figure shows that it is much more difficult to obtain the real cause of insanity in Assam than in England, and when the causes are unknown in two-thirds of the patients, the usefulness of the table is much lessened. Of the deaths 2 were owing to dysentery, 1 to kalá-ázár, 5 to diarrhœa, and 5 to consumption. Of the 31 admissions, 12 were natives of Assam, 15 were imported coolies, and 4 were foreigners. As to religion, 23 were Hindus, 1 was Muhammadan, and 7 were of other castes. Among the year's admissions were 7 criminal lunatics, and this number made a total of 34 of this class under treatment during the year. Not fewer than 14 of these lunatics had committed murder. A new hospital has been built for the female patients, and a similar addition is to be provided for the men. Colonel Carr-Calthrop, M.D., I.M.S., is the principal medical officer and sanitary commissioner, Assam. In his report of the asylum, addressed to the chief commissioner, he says that the high mortality was due chiefly to diarrhœa and dysentery, but that there was also a general increase all round. And Colonel Carr-Calthrop adds the following to his report:—"The superintendent notes that the past year was a generally unhealthy one, and that the unhealthiness of the asylum was shared by the jail and the free population of the district and town of Tezpur, in which diarrhœa and dysentery were very prevalent during the whole of the summer. I have no doubt that this explanation is virtually the correct one." On the other hand, the proceedings of the Chief Commissioner state:—"The most important feature of the year's administration is the excessive mortality of the inmates of the asylum, of which no satisfactory explanation has been furnished. . . . The Chief Commissioner has no sufficient reason for thinking that the year was an unusually unhealthy one, either in the Darrang district or in the town of Tezpur."

